# Assessment of metal residues in soil and evaluate the plant accumulation in copper mine tailings of Dongchuan, Southwest China

**DOI:** 10.3389/fpls.2025.1528723

**Published:** 2025-02-24

**Authors:** Lijuan Deng, Min Yin, Shuanglin Yang, Xiaoyun Wang, Juan Chen, Deren Miao, Genshen Yin, Shuhua Zhai, Yuan Su, Cheng Wu, Zhen Ren

**Affiliations:** ^1^ College of Agronomy and Life Sciences, Kunming University, Kunming, China; ^2^ School of Medicine, Yunnan University, Kunming, China

**Keywords:** copper tailings, metals, phytoremediation, *Salix balfouriana*, combined pollution index

## Abstract

**Introduction:**

This study aimed to identify suitable plants for remediating metal pollution in copper tailing soil and supporting ecological reclamation in Dongchuan, Yunnan, focusing on three major mining regions: Tangdan, Yinmin, and Lanniping.

**Methods:**

The Nemerow comprehensive pollution index was employed to evaluate the metal contamination levels, and the enrichment and transfer capacities of the dominant plants for Cd, Cu, Pb, and Zn were analyzed to identify remediation candidates.

**Results:**

The findings revealed severe pollution in the copper tailing soil, with Cu as the primary pollutant, and with a pollution rate of 77.778%. A total of 96 plant species from 42 families and 87 genera were recorded, including 29 dominant species across 17 families and 26 genera, with herbs comprising 62.068% of the dominant types. Among the tested plants, *Leucaena leucocephala*, *Rumex acetosa*, *Festuca rubra*, and *Salix balfouriana* exhibited significantly higher metal enrichment and transfer capacities, rendering them for ecological restoration. *Salix balfouriana* with the highest comprehensive membership function score of 5.298 was identified as the preferred species for ecological restoration in the Dongchuan Cu tailing area. Correlation analysis revealed a positive relationship between the metal content in underground plant parts and both the total metal content and organic matter (OM) in the rhizosphere soil, whereas a negative correlation was observed with soil pH.

**Discussion:**

The soil in the Dongchuan Cu tailing area is severely contaminated by metals, mainly Cu. Among the identified plants, S. balfouriana emerged as the most suitable candidate for metal accumulation. This study establishes a comprehensive theoretical framework for the application and promotion of phytoremediation technology in the Dongchuan copper tailings area.

## 1 Introduction

Dongchuan District is situated in the northern part of Kunming City, Yunnan Province and is one of the major copper (Cu) mining regions in China. It serves as a key production base, with Cu reserves exceeding 5 million tons, ranking third nationally (Tang et al., 2024). The main Cu mining sites are located in Tangdan, Yinmin, Lanniping, and Luoxue. Copper mining and processing have resulted in substantial solid waste accumulation, leaving significant amounts of Cu tailings in these areas (Tan et al., 2023; Liu et al., 2024). Currently, stockpiling is the primary method for managing tailings. Related studies have shown that zinc (Zn), lead (Pb), and cadmium (Cd) often coexist with Cu in copper tailings, causing severe metal compound pollution in soil (Zhang et al., 2021). Rainwater washes metals into farmlands and rivers, posing a serious threat to local ecosystems. Cheng et al. (2018) reported that the levels of As, Cu, and Zn in the farmland soils around the Dongchuan mines were significantly higher than Yunnan’s background levels, exceeding China’s national risk screening standards. The Xiaojiang River, which flows primarily through Dongchuan, contains sediments with metal concentrations (Cu, Zn, As, Pb, and Cd) that significantly exceed the national Class I standards, with Cu and Cd as the primary pollutants (Huang et al., 2018). Pollution from copper tailings has become the most critical environmental issue in Dongchuan and can severely affect local living conditions. Therefore, developing suitable, cost-effective, and efficient techniques for remediating metal pollution in mining areas in this region is an urgent priority.

Traditional methods for remediating metals in soil include engineering, physical remediation, and chemical remediation. These techniques are often expensive, disrupt the physical and chemical properties of the soil, and pose risks of secondary pollution (Hazrat et al., 2013; Feng and Cheng, 2023; Hu et al., 2024). Phytoremediation is a cost-effective and environmentally friendly approach with remarkable ecological benefits and is used worldwide for the remediation of contaminated or degraded soils. Under non-metallic pollution conditions, the concentrations of Cd, Cu, Pb, and Zn in normal plants are typically within the ranges of 0.05 mg/kg–0.2 mg/kg, 0.40 mg/kg–45.80 mg/kg, 0.10 mg/kg–41.70 mg/kg, and 1 mg/kg–160 mg/kg, respectively (Gerber et al., 2002). In metal-polluted soils, certain plants can naturally colonize and exhibit remarkable tolerance to metal pollution (Hesami et al., 2018). These plants often accumulate higher levels of metals compared to those found in normal plants. To mitigate the risks associated with invasive species, phytoremediation research has focused on screening for native plants in polluted areas. Marjorie and Virginia (2018) demonstrated the effectiveness of *Paspalum thunbergii*, a native Philippine plant, in improving Cu tailings. Similarly, Elizabeth et al. (2017) identified three native Chilean plants, *Prosopis tamarugo*, *Schinus molle*, and *Atriplex nummularia*, which can mitigate metal pollution with strong resistance to Cu and Pb. Dong et al. (2024) discovered that *Kalidium foliatum* and *Graciles* were dominant in tailing ponds, exhibiting the high tolerance to Cd, Cu, Pb, and Zn, with particularly strong Cd accumulation. These findings highlight the exceptional adaptive abilities and metal tolerance of plants established naturally in metal-contaminated mining areas, rendering them ideal candidates for vegetation restoration and ecological rehabilitation.

Analyzing the characteristics of metal pollution in copper tailing soil and identifying suitable pollution-tolerant plant species is essential for vegetation restoration and ecological management in mining areas. In this study, Dongchuan copper tailings were used to assess the metal accumulation capacity of the predominant plant species in the area, with the aim of identifying native plants that can thrive in the copper tailing environment and have a strong ability for metal enrichment. These results provide a basis for utilizing native plants in the ecological restoration of the Dongchuan tailing area and offer theoretical support for managing complex soil metal pollution.

## 2 Materials and methods

### 2.1 Sampling sites

**Figure 1:**
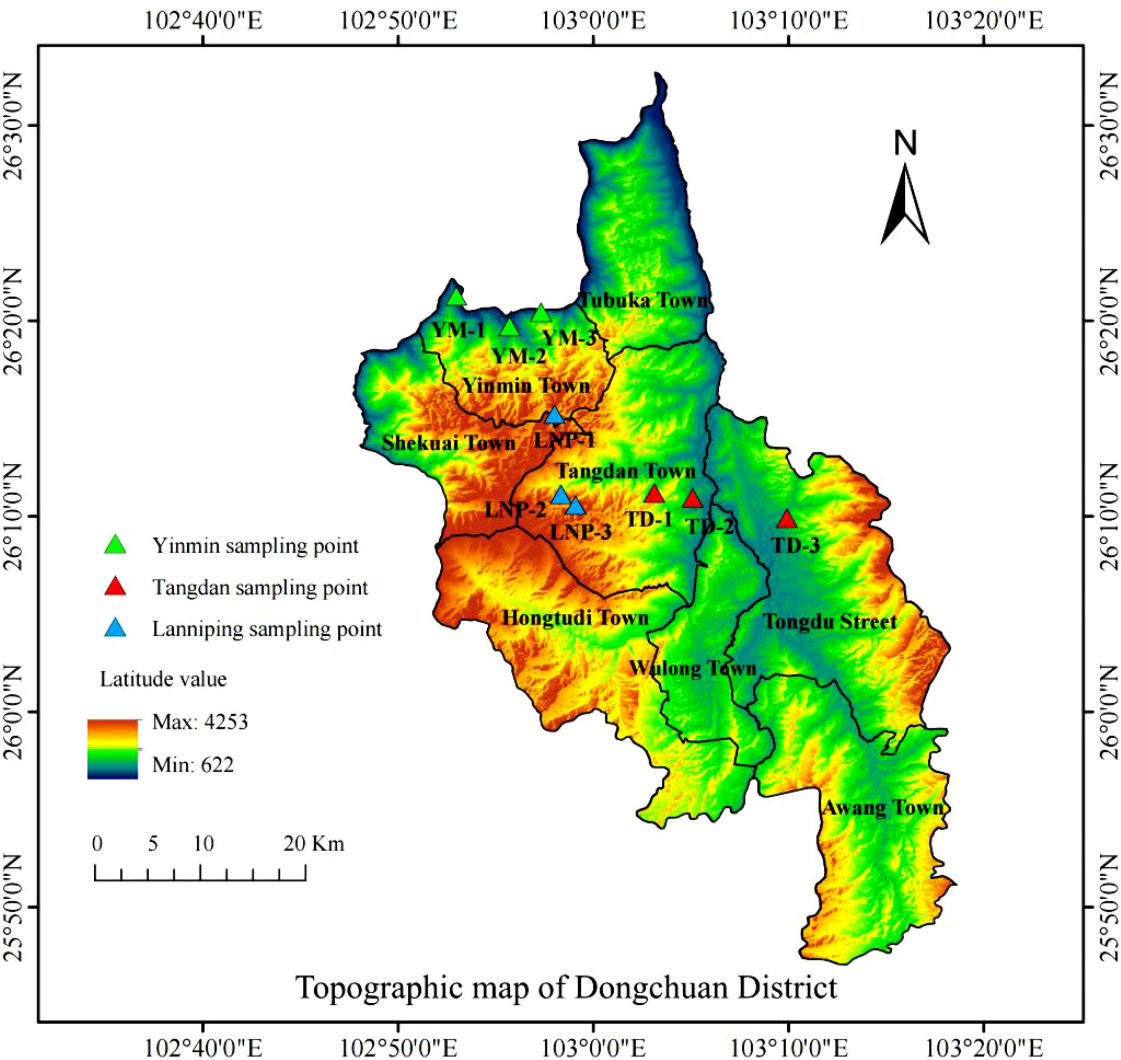
Schematic diagram of the sampling sites of Dongchuan copper tailings.

The study area was situated in Dongchuan District, Kunming City, Yunnan Province, and samples were collected from three representative copper tailing sites: Yinmin, Tangdan, and Lanniping (**Figure 1**). In the Yinmin area, the sampling points were located at coordinates 102°52′58.001″E–102°57′18.846″E, 26°19′43.764″N –26°21′16.914″N, with the elevations ranging from 892.14 m to 924.03 m, labeled YM-1, YM-2, and YM-3. In Tangdan, the sampling points were positioned at 103°3′6.737″E to 103°5′3.741″E and 26°10′56.802″N to 26°11′11.705″N, with the elevations between 1591.27 m and 1963.49 m, labeled TD-1, TD-2, and TD-3. In Lanniping, the sampling points were situated at 102°58′3.317″E to 102°59′5.379″E and 26°15′14.901″N to 26°10′34.989″N, with the elevations ranging from 2,881.27 m to 2,931.18 m, labeled LNP-1, LNP-2, and LNP-3.

### 2.2 Sampling methods

Sampling surveys were conducted in three major copper tailing areas in Dongchuan between May and July 2023. At each site, five 10 m × 10 m plots were randomly established, and the plant types within each plot were recorded. Surface soil, dominant plant, and rhizosphere soil samples were collected. The surface soil was sampled using the five-point method, which involves the removal of surface debris and collection of soil from a depth of 0 cm to 20 cm. For dominant plants and rhizosphere soil, at least four individuals of each dominant species were randomly selected and collection methods were tailored to the plant type. For larger plants (trees and shrubs), samples were collected from the stems and leaves in four cardinal directions, and the roots were sampled within the 0 cm–50 cm horizontal range and 0 cm–30 cm soil depth, with rhizosphere soil collected from the root-attached soil. For smaller plants (herbaceous species), whole plants with soil were extracted and the root-adhering soil was collected as the rhizosphere soil. The 29 plants and their corresponding soil samples were collected, as well as nine surface soil samples, with three replicates for each sample. The samples were sealed separately and transported to the laboratory for further analysis. The surface soil samples were tested for pH, electrical conductivity, organic matter, total nitrogen, total phosphorus, total potassium, DTPA-extractable concentrations, and the pseudo-total concentrations of Cu, Pb, Zn, and Cd were measured in both plant and rhizosphere soils.

### 2.3 Sample processing and analysis

After air-drying and debris removal, the soil samples were homogenized using the quartering method, and 1 kg was retained for analysis. The sample was ground using a mortar and pestle and sieved through a 2 mm mesh. The soil electrical conductivity (EC) and pH were measured using a conductivity meter and pH meter, respectively (Cheng et al., 2024), with a soil-to-water ratio of 1:5. The soil organic matter (OM) content was determined using the potassium dichromate oxidation method with external heating (Lala and Kuldeep, 2024), and total nitrogen (N) was measured using the Kjeldahl method (Xue et al., 2024). The total phosphorus (P) content was analyzed using the molybdenum blue colorimetric method (Taylor, 2000) and the total potassium (K) content was measured using flame photometry. The metal concentrations (Cd, Cu, Pb, and Zn) were determined after soil digestion with *aqua regia* using inductively coupled plasma optical emission spectrometry (ICP-OES). Diethylene-triamine-pentaacetic acid (DTPA) was used as the leaching agent to assess metal content in the exchangeable fraction, following procedures identical to those used for total content determination. All analyses were conducted in triplicate, and reagent blanks were used to correct the determinations. Standard soil and plant reference materials (National Research Center for Standards of China, GBW07405 and GBW07602, respectively) were used for quality assurance.

Plant samples were washed with tap water, separated into aboveground and belowground parts, and rinsed three to five times with ultrapure water. The samples were placed in paper envelopes, deactivated at 105°C for 30 min, and dried at 80°C to a constant weight. Once dried, the samples were ground to powder and sieved through a 100-mesh nylon sieve. Metals (Cd, Cu, Pb, and Zn) in the plant parts were extracted using the hydrogen peroxide-nitric acid digestion method and analyzed using ICP-OES (Liu et al., 2024).

### 2.4 Data analysis

#### 2.4.1 Soil pollution assessment

The single-factor pollution index was calculated using the risk screening values for agricultural land soil pollution from the “Soil environment quality risk control standard for soil contamination of agriculture land (GB15628-2018)” as the evaluation standard for metals content in the soil of the study area.


$${\text{P}}_{\text{i}}{\text{=C}}_{\text{i}}{\text{/S}}_{\text{i}}$$

where P_i_ represents the single-factor pollution index for metal I, C_i_ represents the measured concentration of metal I, and S_i_ represents the maximum allowable limit for metal i, which in this study was the soil environmental risk-screening value. P_i_ ≥1 indicates that the soil is polluted by metal i, with higher values reflecting greater pollution levels. Conversely, if P_i_ <1, the concentration of metal i is within safe limits, indicating no pollution.

A comprehensive pollution index, also known as the Nemerow pollution index (Kumi et al., 2023), can be adopted to assess the study area as a whole and provide a holistic reflection of the impact of various metals on the soil quality. It is particularly suitable for evaluating soils contaminated with multiple metals.


$${\text{P}}_{\text{N}}=\sqrt{{\text{P}}_{\text{avg}}2^{\;}+{\text{P}}_{\text{max}}2^{\;}/2}$$

where P_N_ represents the comprehensive pollution index, P_avg_ represents the average value of pollution indices for Cd, Cu, Pb, and Zn in the soil, and P_max_ represents the maximum pollution index among all metals. The soil pollution grade classifications are presented in **Table 1**.

**Table 1:** Pollution grade classification.

Grade division	P_com_	Class of pollution
I	0–0.7	Safety level
II	0.7–1	Alert level
III	1–2	Mild pollution
IV	2–3	Moderate pollution
V	>3	Heavy pollution

#### 2.4.2 Phytoremediation potential of plants

The bioaccumulation factor (BF) is used to assess the efficiency of metal accumulation in plants (Ravanbakhsh et al., 2016). The BF of aboveground plant parts was calculated as follows:


$${\text{BF=C}}_{\text{Aerial}}{\text{/C}}_{\text{Soil}}$$

The translocation factor (TF), the ability of plants to translocate metals from roots to shoots was defined as follows (Siyar et al., 2022):


$${\text{TF=C}}_{\text{Aerial}}{\text{/C}}_{\text{Root}}$$

#### 2.4.3 Membership function analysis for screening candidate remediation plants

The membership function quantifies the fuzzy concept of the research subject (Li et al., 2024). In this study, the enrichment coefficients for Cu, Cd, Pb, and Zn were used as the evaluation criteria to identify the optimal native plants for remediating Cu tailings in Dongchuan.


$$\text{R}\left({\text{X}}_{\text{i}}\right){\text{ = (X}}_{\text{i}}{\text{-X}}_{\text{min}}\text{)/}\left({\text{X}}_{\text{max}}{\text{-X}}_{\text{min}}\right)$$

where R(X_i_) represents the membership value for metal accumulation capability in a sample; X_i_ represents the measured value for the indicator; and X_min_ and X_max_ represent the minimum and maximum values of a certain indicator for all dominant plants, respectively. The membership function values for each screening indicator were summed to calculate comprehensive membership function values.

#### 2.4.4 Chart creation and data analysis

Sampling point maps were created using ArcGIS 10.8, and figures were generated using Origin (2022). Data organization and calculations were conducted in Excel, whereas single-factor variance, correlation, and cluster analyses were performed using SPSS 26.0. One-way ANOVA was performed to assess the significance of the differences between means. Differences between individual means were tested using the least significant difference (LSD) test. Data are shown in figures and tables as mean ± standard deviation (n = 3).

## 3 Results

### 3.1 Soil physicochemical properties in study area

The physicochemical properties of the soils in the study area are summarized in **Table 2**. Soil pH ranged from 7.763 to 9.383, indicating an alkaline tendency. The soil EC values varied between 0.360 dS/m and 1.920 dS/m, with YM-3 presenting a significantly higher EC than the other sampling points. The soil nutrient content was as follows: total N, 0.120 g/kg–3.070 g/kg; total P, 0.040 g/kg–4.187 g/kg; total, K 6.533 g/kg–16.950 g/kg; and OM, 8.630 g/kg–31.767 g/kg. Although the average nutrient content exhibited no significant differences among the three sampling areas, notable variations were observed at the individual points. The Lanniping area had the highest total N and P levels, with the total N in LNP-1 significantly exceeding that at the other points. The total P contents in the LNP-2 and TD-3 soils were similar, but higher than those at other locations. The maximum total K and OM contents were identified in the Tangdan area, with TD-3 demonstrating the highest total K and TD-1 the highest OM contents. Overall, the Dongchuan copper tailing soils were alkaline and nutrient-poor, with significant variability between the sampling points.

**Table 2:** Analysis of soil properties in the study area.

Sampling sites	pH	EC (dS/m)	Total N (g/kg)	Total P (g/kg)	Total K (g/kg)	OM (g/kg)
YM-1	8.390 ± 0.151bc	1.457 ± 0.289b	0.233 ± 0.058e	0.273 ± 0.012e	10.767 ± 0.875de	12.42 ± 0.871e
YM-2	9.383 ± 0.061a	0.733 ± 0.055d	0.700 ± 0.200d	0.293 ± 0.093e	12.907 ± 2.325bc	8.630 ± 1.434f
YM-3	7.837 ± 0.291d	1.920 ± 0.090a	1.200 ± 0.300c	0.680 ± 0.026c	11.260 ± 0.193cd	22.757 ± 1.732d
Average	8.537 ± 0.780A	1.370 ± 0.600A	0.710 ± 0.485A	0.413 ± 0.231A	11.647 ± 1.121A	14.603 ± 7.314A
TD-1	8.440 ± 0.262bc	0.633 ± 0.061de	1.533 ± 0.153bc	0.413 ± 0.051d	6.533 ± 0.280g	31.767 ± 1.827a
TD-2	8.003 ± 0.25cd	1.153 ± 0.180c	1.567 ± 0.351bc	1.043 ± 0.006b	7.610 ± 0.017fg	12.993 ± 2.152e
TD-3	8.223 ± 0.359bcd	1.153 ± 0.060c	0.120 ± 0.010e	4.187 ± 0.093a	16.950 ± 1.282a	22.380 ± 0.658d
Average	8.220 ± 0.220A	0.977 ± 0.300B	1.073 ± 0.826A	1.880 ± 2.025A	10.363 ± 5.730A	22.380 ± 9.390A
LNP-1	8.117 ± 0.035bcd	0.360 ± 0.046e	3.070 ± 0.405a	0.097 ± 0.025f	8.983 ± 0.404ef	24.463 ± 1.504cd
LNP-2	7.763 ± 0.341d	0.500 ± 0.265de	1.693 ± 0.205b	4.257 ± 0.015a	12.567 ± 1.616bcd	26.170 ± 1.508bc
LNP-3	8.580 ± 0.399b	0.677 ± 0.035d	1.687 ± 0.067b	0.040 ± 0f	13.817 ± 0.379b	27.310 ± 0.570b
Average	8.153 ± 0.411A	0.513 ± 0.160A	2.150 ± 0.797A	1.467 ± 2.419A	11.790 ± 2.513A	25.980 ± 1.434A

Lowercase letters indicate comparisons among all sites in the study area. Uppercase letters indicate comparisons among the three sampled areas. Different letters indicate significant differences.

As shown in **Table 3**, the overall content of metals in the soil of the Dongchuan copper tailings area followed the order Cu > Zn > Pb > Cd. The analysis results of the total amount of metal elements indicate that the pseudo-total metal of the soil in sampling area TD is conspicuously higher than that of other sampling areas. The contents of Cd, Cu, and Zn in the soil at sampling point TD-3 were the highest, attaining 3.950 mg/kg, 6,811.447 mg/kg, and 500.977 mg/kg, respectively, which is significantly higher than that of other sampling points, and the Pb content in the soil at sampling point TD-1 was as high as 243.260 mg/kg, which is significantly higher than that of other sampling points. Among the nine sampling points, the order of the DTPA extractable concentrations of the four metal elements is in accordance with the order of the total metal content in the soil, namely Cu > Zn > Pb > Cd. Among them, the concentrations of DTPA-Cd, DTPA-Cu, and DTPA-Zn in the soil at sampling point TD-2 were the highest and significantly higher than those at other sampling points. The concentration of DTPA-Pb in the soil at sampling point TD-1 was the highest, and the disparity with the other sampling points was significant. Considering all the analyzed soil samples, the DTPA extractable concentrations of Cd, Cu, Pb, and Zn account for 4.694%, 1.991%, 1.973%, and 2.128% of the corresponding total metal concentrations, respectively.

**Table 3:** The total and available contents of metals in soil.

Sampling sites		Cd (mg/kg)	Cu (mg/kg)	Pb (mg/kg)	Zn (mg/kg)
YM-1	Total	0.300 ± 0.173g	185.617 ± 5.010e	20.923 ± 6.657e	102.773 ± 5.174d
	DTPA	0.010 ± 0D	7.497 ± 2.566F	0.450 ± 0.026C	0.993 ± 0.345D
YM-2	Total	0.063 ± 0.012fg	467.420 ± 24.951e	7.950 ± 1.205e	70.547 ± 8.931d
	DTPA	0.023 ± 0.015CD	28.017 ± 0.724DE	1.280 ± 2.087BC	0.840 ± 0.581D
YM-3	Total	0.383 ± 0.074efg	1,702.580 ± 44.173d	23.560 ± 5.005e	105.567 ± 45.486d
	DTPA	0.027 ± 0.006CD	28.717 ± 5.031DE	0.870 ± 0.161BC	1.807 ± 0.813CD
TD-1	Total	2.207 ± 0.185c	4,224.403 ± 208.265b	243.260 ± 29.865a	174.803 ± 13.508c
	DTPA	0.073 ± 0.006B	45.740 ± 3.100C	4.953 ± 0.199A	4.827 ± 0.175BCD
TD-2	Total	0.850 ± 0.087d	4,055.110 ± 82.018b	73.143 ± 11.795c	184.517 ± 6.108c
	DTPA	0.323 ± 0.064A	174.900 ± 2.744A	0.737 ± 0.122BC	9.840 ± 1.280A
TD-3	Total	3.950 ± 0.408a	6,811.447 ± 526.382a	132.187 ± 9.424b	500.977 ± 28.621a
	DTPA	0.067 ± 0.015BC	26.557 ± 0.536DE	1.210 ± 0.485BC	7.610 ± 5.666AB
LNP-1	Total	0.707 ± 0.040de	202.373 ± 19.375e	55.953 ± 2.843cd	159.657 ± 13.828c
	DTPA	0.037 ± 0.029BCD	31.207 ± 0.603D	2.147 ± 1.493B	5.757 ± 3.021BC
LNP-2	Total	0.570 ± 0.085def	3,399.397 ± 658.997c	49.400 ± 12.669d	245.193 ± 48.071b
	DTPA	0.010 ± 0D	79.920 ± 0.831B	0.203 ± 0.006C	1.573 ± 0.021CD
LNP-3	Total	3.537 ± 0.352b	1,411.767 ± 210.647d	13.970 ± 3.143e	99.050 ± 12.308d
	DTPA	0.020 ± 0D	24.720 ± 2.560E	0.390 ± 0.147C	1.733 ± 0.387CD

In the same column, different lowercase letters indicate significant differences in the pseudo-total metal content among different sample points (p <0.05), while different uppercase letters indicate significant differences in the concentration of DTPA-extractable among different sample points (p <0.05).

### 3.2 Assessment of soil contamination

The metal pollution indices for Dongchuan copper tailings are presented in **Table 4**. The soil pH at all sampling points exceeded 7.5. The risk-screening values for Cd, Cu, Pb, and Zn were 0.6 mg/kg, 100 mg/kg, 170 mg/kg, and 300 mg/kg, respectively. The single-factor pollution index values for Cd, Cu, Pb, and Zn ranged from 0.500 to 6.583, 1.856 to 68.115, 0.047 to 1.431, and 0.235 to 1.670, respectively, indicating pollution severity in the order Cu > Cd > Pb > Zn. Copper was the most severe pollutant, with a heavy pollution rate of 77.778%. On average, Pb and Zn concentrations in the soil were within national risk control limits. The comprehensive pollution index (Nemerow index) ranged from 1.405 to 50.058 across all sampling points with an average of 18.349, indicating heavy pollution. These results indicate severe composite metal pollution in the Dongchuan copper tailing area, with Cu as the primary pollutant.

**Table 4:** Soil pollution index in the study area.

Sampling sites	Single pollution index				P_N_	Pollution level
	Cd	Cu	Pb	Zn		
YM-1	0.500 ± 0.289	1.856 ± 0.050	0.123 ± 0.039	0.343 ± 0.017	1.405 ± 0.056	Mild pollution
YM-2	0.106 ± 0.019	4.674 ± 0.250	0.047 ± 0.007	0.235 ± 0.030	3.424 ± 0.180	Heavy pollution
YM-3	0.639 ± 0.123	17.026 ± 0.442	0.139 ± 0.029	0.352 ± 0.152	12.460 ± 0.316	Heavy pollution
TD-1	3.678 ± 0.308	42.244 ± 2.083	1.431 ± 0.176	0.583 ± 0.045	31.050 ± 1.519	Heavy pollution
TD-2	1.417 ± 0.144	40.551 ± 0.820	0.430 ± 0.069	0.615 ± 0.020	29.665 ± 0.600	Heavy pollution
TD-3	6.583 ± 0.680	68.115 ± 5.264	0.778 ± 0.055	1.670 ± 0.095	50.058 ± 3.807	Heavy pollution
LNP-1	1.178 ± 0.067	2.024 ± 0.194	0.329 ± 0.017	0.532 ± 0.046	1.601 ± 0.135	Mild pollution
LNP-2	0.950 ± 0.142	33.994 ± 6.590	0.291 ± 0.075	0.817 ± 0.160	24.868 ± 4.815	Heavy pollution
LNP-3	5.894 ± 0.587	14.118 ± 2.106	0.082 ± 0.018	0.330 ± 0.041	10.618 ± 1.492	Heavy pollution
Average	2.327 ± 2.366	24.956 ± 21.992	0.406 ± 0.433	0.609 ± 0.426	18.350 ± 16.115	Heavy pollution

### 3.3 Floristic composition in the study area

A total of 42 families, 87 genera, and 96 plant species were identified in the study area (**Table 5**), including 58 species in Tangdan, 50 species in Yinmin, and 38 species in Lanniping. Herbaceous plants were dominant, comprising 72 species (75%), followed by shrubs with 14 species (14.583%), and five species of trees and lianas (5.208%). Among the 29 dominant species, representing 17 families and 26 genera, Asteraceae was the most common family with six species (20.689%), followed by Poaceae and Polygonaceae, each with four species (13.793%), and Malvaceae with two species (6.896%). Other families, including Euphorbiaceae, Apocynaceae, Hypericaceae, Acanthaceae, Loganiaceae, Coriariaceae, Equisetaceae, Cyperaceae, Scrophulariaceae, Urticaceae, Salicaceae, and Bignoniaceae, were represented by one species each. Herbaceous plants accounted for 18 dominant species (62.068%), followed by shrubs (27.586%) and only two tree species, *Salix balfouriana* and *Leucaena leucocephala*.

**Table 5:** List of plant species growing on the Dongchuan copper tailings in Yunnan, China.

Family	Species	Abundance			Altitude (m)	Life form
		TD	YM	LNP		
Acanthaceae	*Barleria cristata* L.	F	D	–	881	Shrub
	*Lepidagathis incurva* Buch.-Ham. ex D. Don	O	–	–	1,618	Perennial grass
Amaranthaceae	*Alternanthera pungens* H.B.K	–	O	–	905	Annual grass
	*Amaranthus blitoides* S. Watson	F	D	–	905	Annual grass
	*Chenopodium album* L.	–	R	–	906	Annual grass
Anacardiaceae	*Pistacia chinensis* Bunge	–	O	–	882	Tree
Asteraceae	*Ageratina adenophora* (Spreng.) R. M. King & H. Rob.	D	F	O	1,958	Perennial grass
	*Ageratina conyzoides* L.	–	–	R	906	Annual grass
	*Anaphalis nepalensis* (Spreng.) Hand.-Mazz.	D	–	O	2,882	Perennial grass
	*Artemisia lavandulaefolia* DC.	D	F	F	2,919	Perennial grass
	*Artemisia dubia* Wall. ex Besser subf. intermedia Pamp.	F	–	D	2,935	Annual grass
	*Aster albescens* (DC.) Wall. ex Koehne	O	–	–	2,871	Shrub
	*Bidens pilosa* L.	R	F	O	900	Annual grass
	*Cirsium arvense* var. *integrifolium* Wimm. & Grab.	–	–	R	936	Perennial grass
	*Crassocephalum crepidioides* (Benth.) S. Moore	R	–	–	1,616	Annual grass
	*Dichrocephala benthamii* C. B. Clarke	R	–	–	2,909	Annual grass
	*Erigeron sumatrensis* Retz.	R	R	–	1,620	Biennial grass
	*Erigeron canadensis* L.	D	F	R	1,958	Annual grass
	*Galinsoga parviflora* Cav.	R	O	–	883	Annual grass
	*Lactuca sibirica* (L.) Benth. ex Maxim.	R	R	–	913	Perennial grass
	*Petasites japonicus* (Siebold & Zucc.) Maxim.	–	–	O	2,919	Perennial grass
	*Picris hieracioides* L.	O	–	O	2,919	Biennial grass
	*Pseudognaphalium affine* (D. Don) Anderb.	R	–	–	2,887	Annual grass
	*Senecioscandens* Buch.-Ham. ex D. Don	R	–	–	1,614	Perennial grass
	*Sonchus wightianus* DC.	R	O	–	913	Perennial grass
	*Taraxacum mongolicum* Hand.-Mazz.	F	F	D	1,602	Perennial grass
Apiaceae	*Torilis japonica* (Houtt.) DC.	–	–	R	2919	Perennial grass
Apocynaceae	*Nerium oleander* L.	–	D	R	914	Shrub
Bignoniaceae	*Incarvillea arguta* (Royle) Royle	D	R	–	1,959	Perennial grass
Boraginaceae	*Cynoglossum amabile* Stapf & Drummond	O	–	–	1,613	Perennial grass
Cannabaceae	*Celtis tetrandra Roxb.*	O	–	–	1,948	Tree
Caprifoliaceae	*Leycesteria formosa* Wall.	–	–	R	2,917	Shrub
Caryophyllaceae	*Dianthus carthusianorum* L.	–	–	F	2,936	Perennial grass
Coriariaceae	*Coriaria nepalensis*	–	D	R	1,964	Shrub
Cyperaceae	*Cyperus rotundus* L.	F	D	–	880	Perennial grass
Equisetaceae	*Equisetum hyemale* L.	R	D	–	880	Perennial grass
Euphorbiaceae	*Euphorbia heterophylla* L.	R	–	–	923	Perennial grass
	*Jatropha curcas* L.	–	D	–	913	Shrub
Geraniaceae	*Geraniumsibiricum* L.	R	–	O	1,967	Annual grass
Gramineae	*Apluda mutica* L.	O	–	–	1,961	Perennial grass
	*Arthraxon hispidus* (Thunb.) Makino	O	R	O	913	Annual grass
	*Cynodon dactylon* (L.) Pers.	R	D	–	928	Perennial grass
	*Festuca rubra* L.	–	D	–	881	Perennial grass
	*Festuca arioides* Lam.	R	O	–	882	Perennial grass
	*Phragmites australis* (Cav.) Trin. ex Steud.	R	D	–	881	Perennial grass
Hydrangeaceae	*Deutzia scabra* Thunb.	R	–	–	2,869	Shrub
Hypericaeae	*Hypericum monogynum* L.	D	–	R	2,926	Shrub
Lamiaceae	*Clinopodium megalanthum* (Diels) C. Y. Wu & S. J. Hsuan ex H. W. Li	–	–	R	2,883	Perennial grass
	*Origanum vulgare* L.	–	–	O	2,877	Perennial grass
	*Salvia japonica* Thunb.	O	–	R	2,879	Perennial grass
Lardizabalaceae	*Holboellia latifolia* Wall.	–	R	–	923	Liana
Leguminosae	*Indigofera pendula* Franch.	–	O	–	2,877	Shrub
	*Leucaena leucocephala* (Lam.) de Wit	D	D	–	888	Tree
Loganiaceae	*Buddleja asiatica* Lour.	R	–	O	2,886	Shrub
Malvaceae	*Malva cathayensis* M. G. Gilbert, Y. Tang & Dorr	D	R	–	1,620	Perennial grass
	*Sida yunnanensis* S. Y. Hu	F	D	–	1,608	Shrub
Moraceae	*Ficus tikoua* Bureau	–	O	–	906	Liana
Nephropteridae	*Nephrolepis cordifolia* (L.) C. Presl	R	O	–	879	Perennial grass
Nyctaginaceae	*Boerhavia diffusa* L.	–	O	–	912	Liana
	*Mirabilis jalapa* L.	–	O	–	910	Annual grass
Onagraceae	*Epilobium blinii* H. Lév.	R	–	F	2,918	Perennial grass
	*Epilobium hirsutum* L.	–	–	O	2,930	Perennial grass
Oxalidaceae	*Oxalis corniculata* L.	O	–	–	1,958	Perennial grass
Papaveraceae	*Argemone mexicana* L.	–	F	–	936	Annual grass
phytolaccaceae	*Phytolacca acinosa* Roxb.	O	–	–	921	Perennial grass
Plantaginaceae	*Plantago depressa* Willd.	R	–	R	2,865	Biennial grass
Poaceae	*Calamagrostis epigejos* (L.) Roth	–	R	–	963	Perennial grass
	*Eleusine indica* (L.) Gaertn.	R	F	–	919	Perennial grass
	*Lolium perenne* L.	–	F	–	1,616	Perennial grass
	*Stipa capillata* L.	R	D	–	928	Perennial grass
Polygonaceae	*Fagopyrum statice* (H. Lév.) H. Gross	O	–	–	921	Perennial grass
	*Fagopyrum dibotrys* (D. Don) Hara	–	R	–	915	Perennial grass
	*Fallopia multiflora* (Thunb.) Haraldson	D	R	–	1,618	Perennial grass
	*Oxyria sinensis* Hems	F	D	O	1,959	Perennial grass
	*Persicaria lapathifolia* (L.) Delarbre	R	R	–	2,877	Perennial grass
	*Rumex hastatus* D. Don	D	D	R	880	Annual grass
	*Rumex acetosa* L.	–	R	D	2,914	Perennial grass
Ranunculaceae	*Anemone vitifolia* Buch.-Ham. ex DC.	–	–	O	2,861	Perennial grass
	*Clematis florida* Thunb.	O	–	–	1,612	Liana
	*Thalictrum delavayi* Franch.	–	–	F	2,869	Perennial grass
Raspberry	*Potentilla vesca* (L.) Scop.	–	–	O	2,879	Perennial grass
	*Rubus rosifolius* Sm.	O	–	–	1,620	Shrub
Rubiaceae	*Galium* sp*urium* L.	R	–	–	887	Perennial grass
	*Rubia mandersii* Collett & Hemsl.	–	O	–	934。	Perennial grass
Salicaceae	*Salix balfouriana C. K. Schneid.*	–	–	D	2,882	Tree
Scrophulariaceae	*Buddleja davidii* Franch.	R	–	D	2,883	Shrub
	*Buddleja officinalis* Maxim.	–	–	D	2,879	Shrub
	*Lindenbergia muraria* (Roxb. ex D. Don) Brühl	–	R	–	878	Annual grass
	*Pedicularis* sp*icata* Pall.	R	–	F	2,881	Annual grass
	*Pedicularis verticillata* L.	–	–	R	2,882	Perennial grass
Solanaceae	*Solanum erianthum* D. Don	–	R	–	936	Tree
	*Solanum nigrum* L.	–	F	–	931	Annual grass
Urticaceae	*Debregeasia squamata* King ex Hook. f.	D	–	–	1,958	Liana
Verbenaceae	*Verbena officinalis* L.	–	R	–	931	Perennial grass
Violaceae	*Viola philippica* Cav.	–	–	R	2,909	Perennial grass
Vitaceae	*Euphorbia humifusa* Willd. ex Schltdl.	O	–	–	929	Annual grass

D, dominant; F, frequent; O, occasional; R, rare; –, not existent.

### 3.4 Metals content in plants in the study area

**Figure 2:**
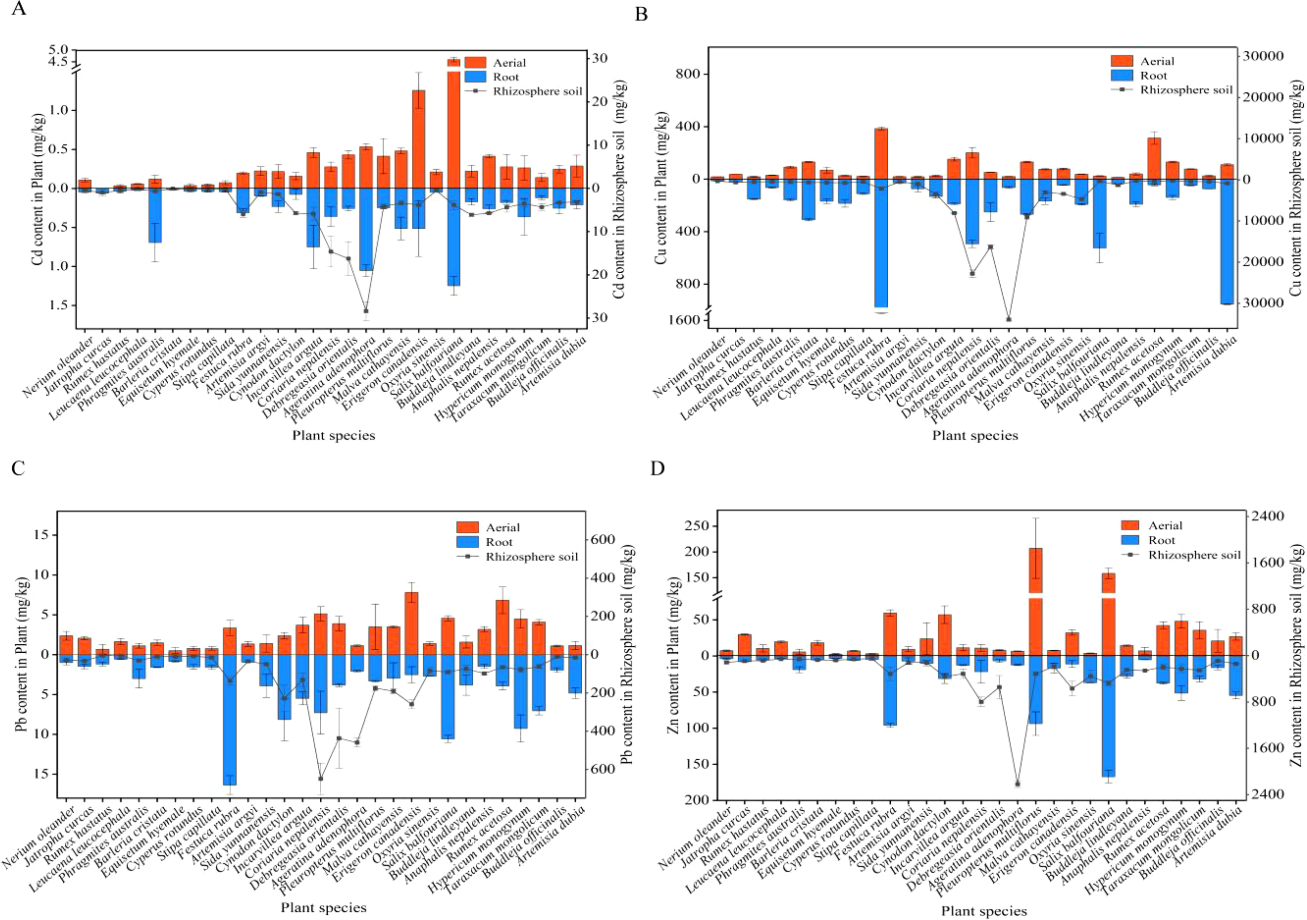
Cadmium, copper, lead, and zinc contents in the different plant tissue parts and rhizosphere soil.

**Figure 2A** illustrates the Cd concentrations in the aboveground and underground parts of the plants in the study area. Fourteen species, including *Sida yunnanensi*, *Incarvillea arguta*, *Coriaria nepalensis*, *Debregeasia orientalis*, *Ageratina adenophora*, *Fallopia multiflora*, *Malva sylvestris*, *Erigeron canadensis*, *S. balfouriana*, *Anaphalis nepalensis*, *Rumex acetosa*, *Hypericum monogynum*, *Buddleja officinalis*, and *Artemisia dubia*, exhibited the Cd concentrations in both parts exceeding those of normal plants. Among them, *S. balfouriana* demonstrated the highest Cd levels in both the aboveground and underground tissues. These findings indicate the presence of several Cd-tolerant plant species in the Dongchuan Cu tailing area, with *S. balfouriana* emerging as a particularly promising candidate for Cd accumulation.

As shown in **Figure 2B**, there are 13 plant species, including *Phragmites australis*, *Barleria cristata*, *Equisetum hyemale*, *Festuca rubra*, *I. arguta*, *C. nepalensis*, *D. orientalis*, *F. multiflora*, *M. sylvestris*, *S. balfouriana*, *H. monogynum*, *Taraxacum mongolicum*, and *A. dubia*, exhibited the Cu levels exceeding the normal range in both parts. Among these, *S. balfouriana* had the highest Cu concentrations in the aboveground parts, whereas *F. rubra* had the highest levels in the underground parts.

As shown in **Figures 2C, D**, *S. balfouriana* exhibited the highest Zn concentrations, reaching 454.628 mg/kg in the aboveground parts and 167.313 mg/kg in the underground parts, both exceeding the normal range. The other surveyed plants maintained their Pb and Zn levels within normal limits.

**Figure 2** highlights the significant variability in the metal content of the rhizosphere soils of the different plants. The Cd, Cu, Pb, and Zn concentrations in the rhizosphere soils of the 29 plant species ranged from 0.080 mg/kg to 28.413 mg/kg, 198.156 mg/kg to 33,968.656 mg/kg, 4.167 mg/kg to 649.630 mg/kg, and 51.640 mg/kg to 2,219.460 mg/kg, respectively. The rhizosphere soil of *A. adenophora* exhibited notably higher levels of Cd, Cu, and Pb than those of other species, suggesting its high tolerance to complex metal pollution. *D. orientalis* had the highest Zn concentration, demonstrating its ability to thrive in Zn-rich environments. Overall, the metal content in native dominant plants and their rhizosphere soils followed the trend Cu > Zn > Pb > Cd.

### 3.5 Plant enrichment coefficients and transfer coefficients in the study area

**Figure 3:**
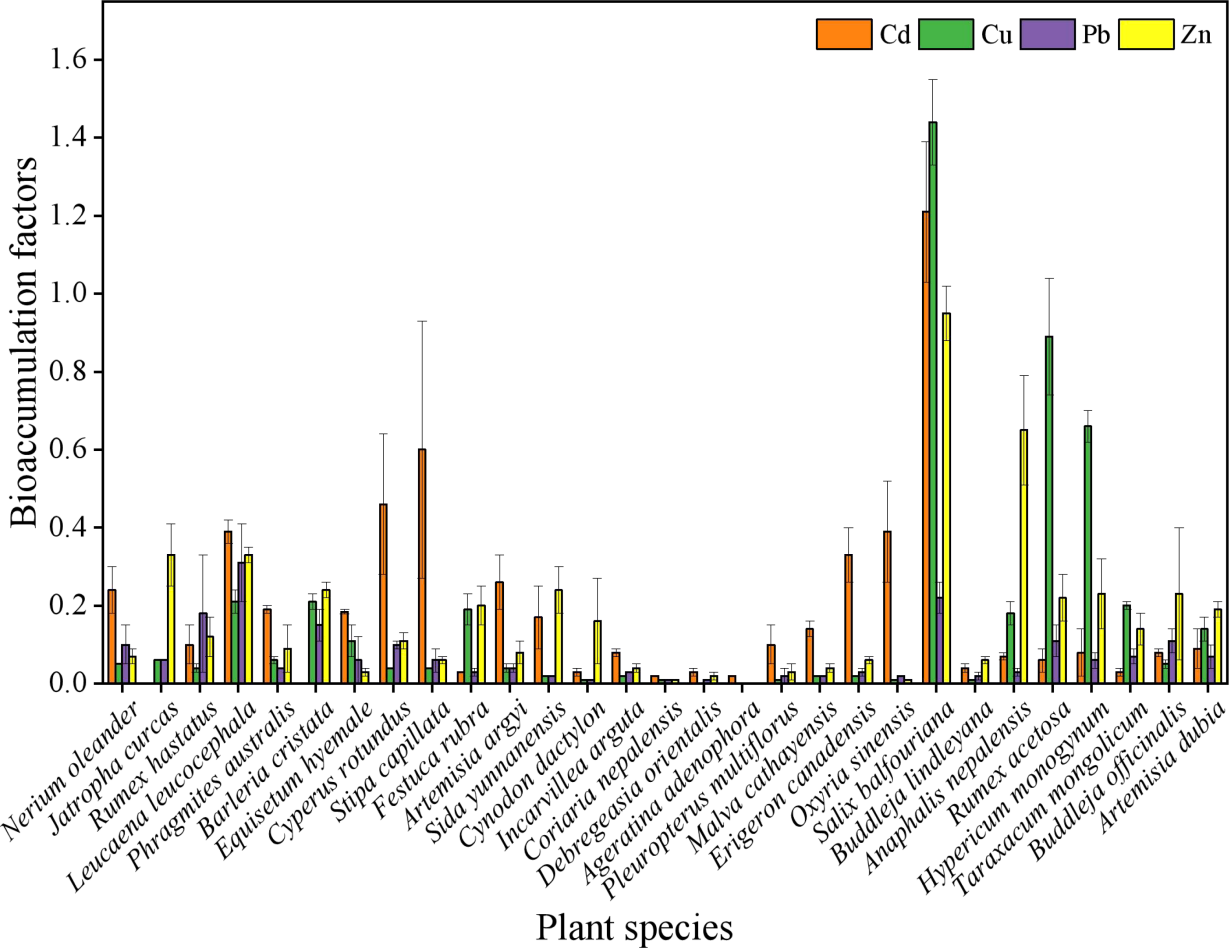
Analysis of enrichment coefficient in dominant plants.

As shown in **Figure 3**, the enrichment coefficients varied significantly among the 29 dominant species. The coefficients for Cd, Cu, Pb, and Zn ranged from 0 to 1.196, 0.007 to 1.431, 0.007 to 0.302, and 0.003 to 0.945, respectively. *S. balfouriana* exhibited the highest enrichment coefficients for Cd, Cu, and Zn, with values exceeding 1 for Cd and Cu. Its enrichment coefficient for Pb (0.221) was second only to that of *Phragmites communis*. None of the plants in the Dongchuan copper tailing area had enrichment coefficients above 1 for all four metals. However, *S. balfouriana* demonstrated a superior overall capacity for metal enrichment compared to the other species.

**Figure 4:**
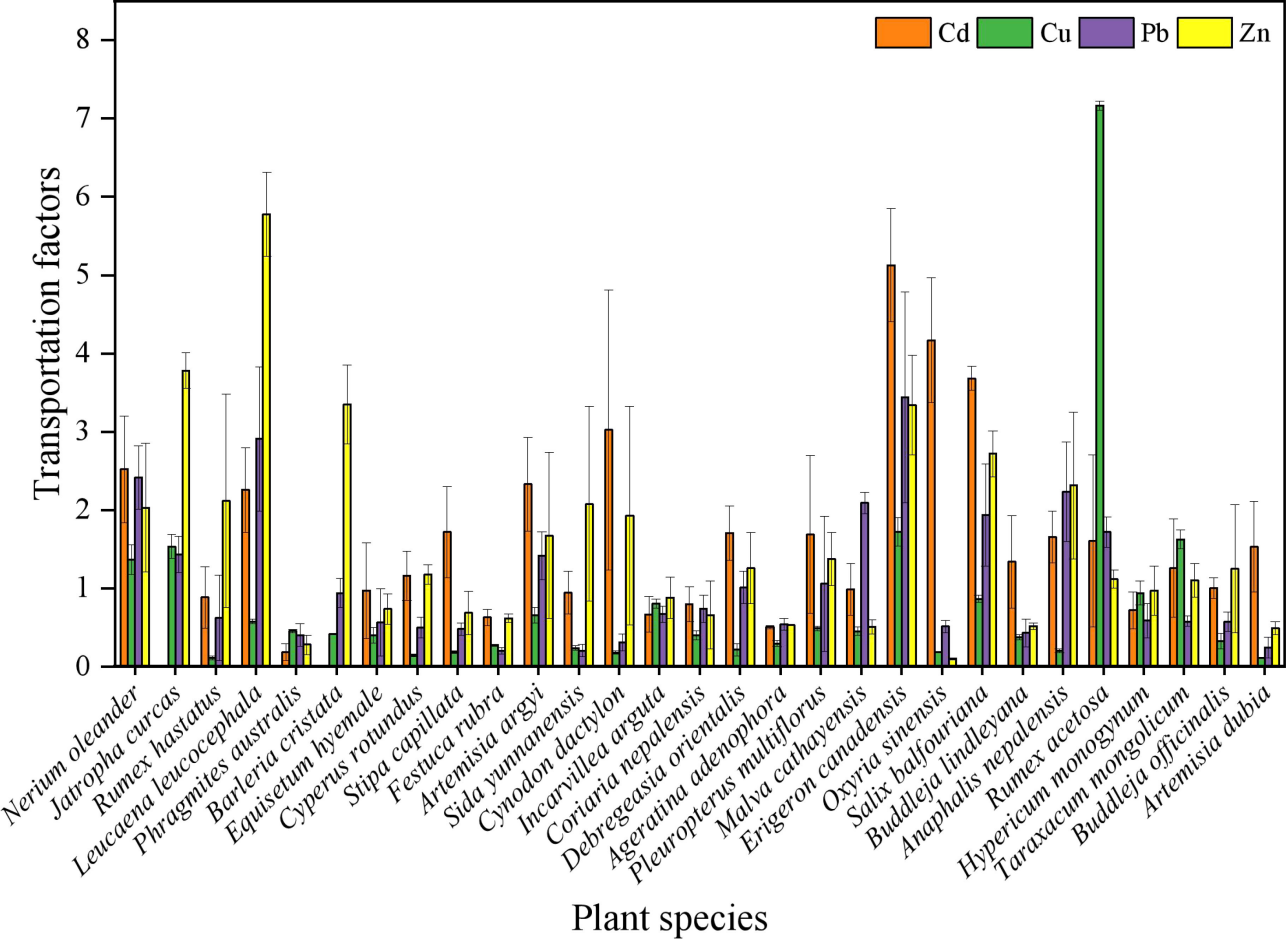
Analysis of the transfer coefficient in the dominant plants.

The transfer coefficient measures the capacity of a plant to transport absorbed elements from the underground to aboveground parts. As shown in **Figure 4**, the transfer coefficients of Cd, Cu, Pb, and Zn in the 29 dominant plants ranged from 0 to 5.129, 0.114 to 7.162, 0.237 to 3.444, and 0.100 to 5.762, respectively. Several plants exhibited transfer coefficients greater than 1 for specific metals. Notably, 17 plants (58.621%) had transfer coefficients exceeding 1 for Cd and Zn, 5 (17.241%) for Cu, and 11 (37.931%) for Pb. These variations reflect the differing ability of plants to transfer various metals. Notably, *Nerium oleander*, *R. acetosa*, and *E. canadensis* exhibited transfer coefficients greater than 1 for all four metals, highlighting their potential as effective candidates for metal extraction and remediation from Cu tailings.

### 3.6 Comprehensive evaluation of enrichment capacity of dominant plants in the study area

#### 3.6.1 Differences between species in metal concentration

**Figure 5:**
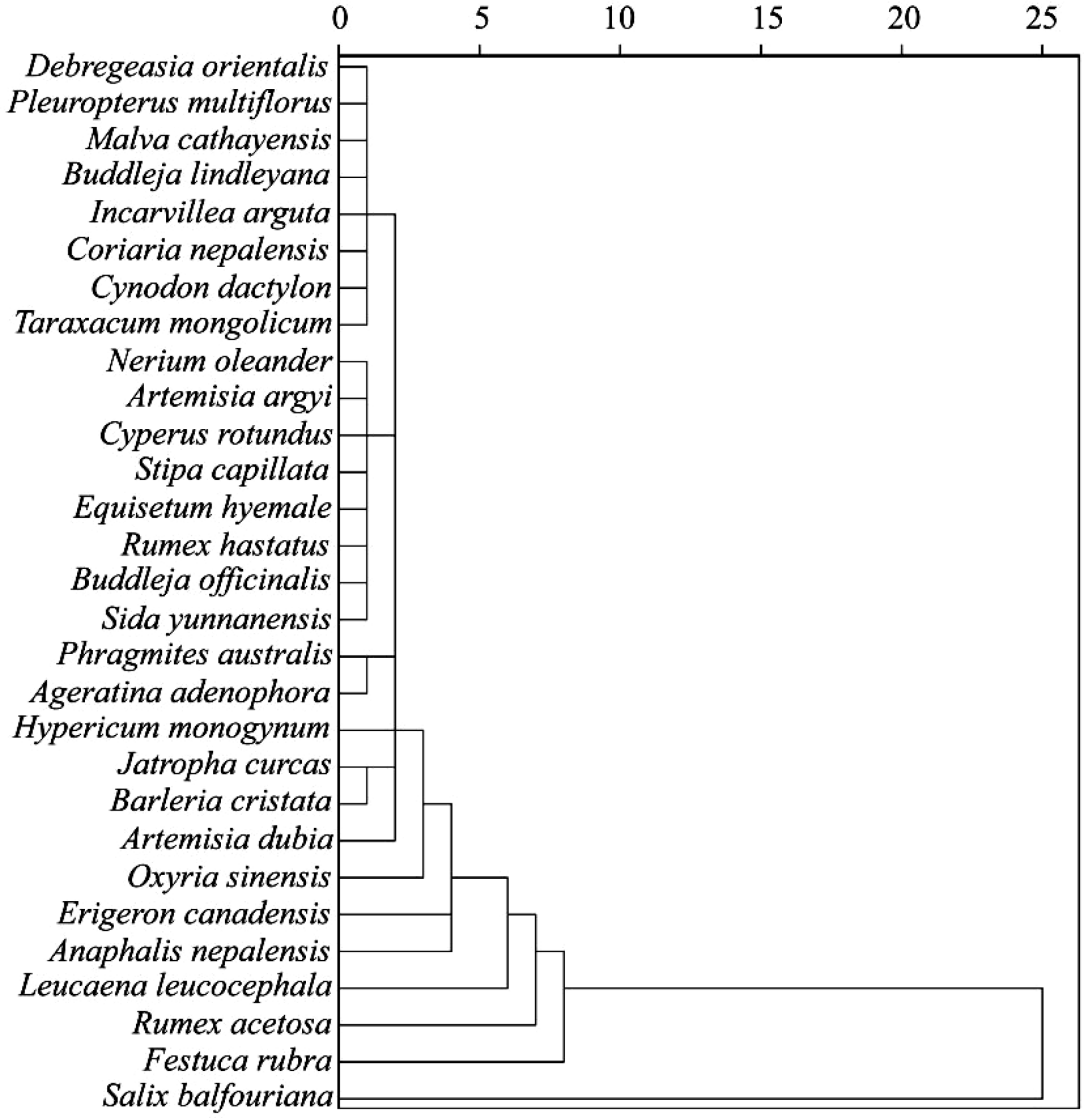
Cluster analysis of enrichment characteristics in dominant plants.

A cluster analysis was performed on the 29 dominant plants using Cd, Cu, Pb, and Zn concentrations in various plant tissues, along with the enrichment and transfer coefficients as parameters. By applying the inter-group average linkage method, the analysis divided the surveyed plants into five clusters (**Figure 5**). (1) Cluster 1: This group included 25 species, such as *D. orientalis*, *F. multiflora*, and *M. sylvestris*, which had the low internal concentrations of Cd, Cu, Pb, and Zn and weak overall metals enrichment capabilities. (2) Cluster 2: This group included *L. leucocephala*, which exhibited the low absorption of Cd, Cu, Pb, and Zn but demonstrated the strong metal extraction capabilities, with the transfer coefficients exceeding 1 for all metals except Cu. (3) Cluster 3: This group included *R. acetosa*, which exhibited transfer coefficients above 1 for Cd, Cu, Pb, and Zn, along with a high enrichment capacity for Cu. (4) Cluster 4: This group included *F. rubra*, which accumulated significant amounts of metals in its underground parts, with Cd, Cu, Pb, and Zn concentrations far exceeding those of the other plants. (5) Cluster 5: This group included *S. balfouriana*, which exhibited high internal concentrations of Cd, Cu, Pb, and Zn, along with strong enrichment coefficients. The aboveground parts demonstrated transfer coefficients greater than 1 for Cd, Pb, and Zn. Among the five types of plants, *S. balfouriana* is an ideal candidate for ecological restoration in areas with complex metal contamination by copper tailings.

#### 3.6.2 Comprehensive analysis of accumulation capacity of dominant plants in the study area

Fuzzy membership function analysis was carried out in this study to evaluate the comprehensive enrichment capacity of different plants for Cd, Cu, Pb, and Zn, with the objective of identifying the optimal candidates for remediating Dongchuan copper tailings. As shown in **Table 6**, the fuzzy membership function scores for the enrichment coefficients of the 29 dominant plants ranged from 0.019 to 5.298. The top five plants in terms of comprehensive extraction capacity were *S. balfouriana*, *A. nepalensis*, *L. leucocephala*, *R. acetosa*, and *H. monogynum*, with comprehensive membership scores of 5.298, 2.397, 1.764, 1.453, and 1.260, respectively. These findings highlight that *S. balfouriana* had the highest overall extraction capacity, making it the most suitable native plant for remediating complex metal pollution in the Dongchuan copper tailing area.

**Table 6:** Comprehensive evaluation of the metal extraction capacity of the dominant plants.

Plant species	Subordinative function value				Total	Rank
	Cd	Cu	Pb	Zn		
*Nerium oleander*	0.185	0.038	0.061	0.201	0.484	20
*Jatropha curcas*	0	0.042	0.044	1.052	1.138	6
*Rumex hastatus*	0.051	0.024	0.113	0.400	0.588	16
*Leucaena leucocephala*	0.322	0.143	0.211	1.088	1.764	3
*Phragmites australis*	0.155	0.041	0.025	0.295	0.516	19
*Barleria cristata*	0	0.146	0.104	0.808	1.057	7
*Equisetum hyemale*	0.192	0.074	0.033	0.100	0.399	15
*Cyperus rotundus*	0.355	0.025	0.067	0.364	0.812	10
*Stipa capillata*	0.383	0.030	0.035	0.185	0.633	13
*Festuca rubra*	0.028	0.126	0.017	0.621	0.791	12
*Artemisia argyi*	0.210	0.025	0.027	0.253	0.515	18
*Sida yunnanensis*	0.137	0.012	0.019	0.663	0.831	8
*Cynodon dactylon*	0.023	0.005	0.007	0.515	0.549	17
*Incarvillea arguta*	0.065	0.013	0.019	0.114	0.211	25
*Coriaria nepalensis*	0.016	0.006	0.005	0.037	0.064	28
*Debregeasia orientalis*	0.022	0.002	0.006	0.041	0.071	27
*Ageratina adenophora*	0.016	0	0.001	0.002	0.019	29
*Pleuropterus multiflorus*	0.083	0.010	0.014	0.083	0.189	26
*Malvasylvestris*	0.119	0.017	0.012	0.127	0.274	23
*Erigeron canadensis*	0.275	0.016	0.021	0.181	0.493	21
*Oxyria sinensis*	0.317	0.005	0.011	0.027	0.361	22
*Salix balfouriana*	1.000	1.000	0.154	3.144	5.298	1
*Buddleja lindleyana*	0.030	0.007	0.015	0.187	0.238	24
*Anaphalis nepalensis*	0.060	0.124	0.022	2.191	2.397	2
*Rumex acetosa*	0.053	0.619	0.072	0.708	1.453	4
*Hypericum monogynum*	0.063	0.460	0.040	0.697	1.260	5
*Taraxacum mongolicum*	0.027	0.142	0.046	0.462	0.677	14
*Buddleja officinalis*	0.063	0.038	0.070	0.767	0.938	9
*Artemisia dubia*	0.076	0.092	0.051	0.626	0.845	11

### 3.7 The impact of soil properties on metal concentrations in plants

**Figure 6:**
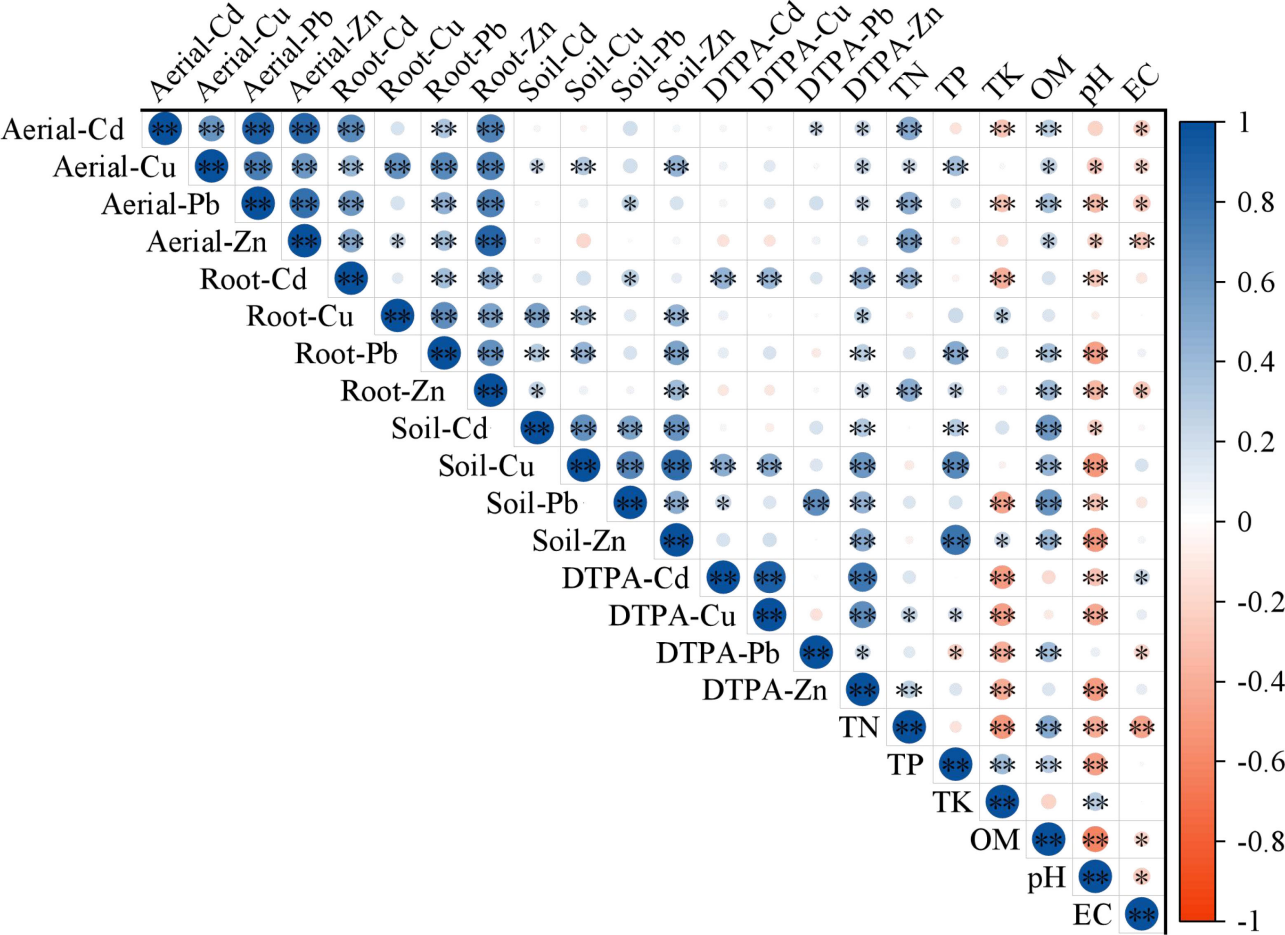
Correlation analysis of plant metal content and soil factors. * indicates *P <*0.05 with significant correlation; ** indicates *P <*0.01 with highly significant correlation.

As shown in **Figure 6**, a significant positive correlation was observed between the metal content in the shoots and roots of the plants. Among them, the content of Cu, Pb and Zn in the root parts of plants was positively correlated with the total content of the corresponding metals in the soil, with the correlation of Cu and Zn being significant. The Cd content in the root parts of the plants was significantly and positively correlated with the DTPA-Cd concentration in the soil. Additionally, soil pH and organic matter content are crucial factors affecting metal content in soil and plants. The soil organic matter content was significantly and positively correlated with the metal content in the soil and plants, whereas soil pH was negatively correlated with the metal content in the soil and plants.

## 4 Discussion

### 4.1 Metal pollution status in Dongchuan copper tailings

This study identified widespread complex metal contamination in the soil of Dongchuan Cu tailings, with Cd, Cu, Pb, and Zn levels generally exceeding the local background values (Lu et al., 2021; Lu et al., 2024). The comprehensive pollution index indicated severe pollution, with Cu as the primary pollutant. Similar findings were reported by Xiao et al. (2022), who observed that the Cd, Cu, Pb, and Zn concentrations in Shirao copper tailings exceeded China’s environmental risk screening values (GB15628-2018), with Cu being the most prevalent. Punia et al. (2017) reported a similar pattern in Khetri Cu tailings in northwestern India, where increased Cd, Cu, Pb, and Zn levels were linked to mining activities and bedrock weathering. These findings suggest that after Cu was extracted from the primary ore via flotation, residual elements accumulated on the surface along with Cu tailings. During ore cracking, these elements are activated and released in ionic or molecular forms into the soil, leading to complex metal pollution (Batista et al., 2006; Wang et al., 2019). Cu is expected to be the dominant pollutant in Dongchuan, accompanied by Cd, Pb, and Zn contamination.

### 4.2 Metal accumulation and translocation capacity of dominant plants in Dongchuan

Based on the enrichment levels and adsorption characteristics of metals in the soil, plants can be categorized into two distinct groups: accumulators and excluders (Baker, 1981). The findings of this study revealed that the majority of the dominant plants in Dongchuan, including those from the Compositae, Gramineae, and Polygonaceae families, fall into the category of excluders. These plants, mostly perennial herbs, are characterized by rapid growth and reproduction and are capable of colonizing contaminated areas within a short period of time (Wu et al., 2021). These plants are suitable for planting in the later stages of bioremediation and can be employed to enhance plant diversity in the mining area and establish a favorable plant community structure (Zhao et al., 2012). Additionally, four plants, *L. leucocephala*, *A. nepalensis*, *R. acetosa*, and *S. balfouriana*, have relatively high metal concentrations in their tissues and can be used as accumulators for ecological restoration in mining areas.

*L. leucocephala* and *S. balfouriana* are the only two tree species in the study area. Like all legumes, *L. leucocephala* is characterized by a large biomass and well-developed root systems that can extract metals from deep soil layers to its aboveground parts (Jean and Khasa, 2022). This plant also has the ability to fix nitrogen, which can restore soil fertility while extracting soil metals, thus benefiting the reclamation of tailings (Nishida and Suzaki, 2018). Adanikin and Kayode (2019) found that using *L. leucocephala* for metal-polluted soil remediation resulted in a 41%, 61.6%, and 52.72% decrease in the total amounts of Zn, Pb, and Cu in the soil, respectively. *S. balfouriana* is known for its rapid growth and large biomass (Kuzovkina and Volk, 2009). Studies have shown that certain clonal varieties of willow trees possess strong abilities to absorb and transfer metals to the aboveground parts (Norini et al., 2019). Therefore, both *L. leucocephala* and *S. balfouriana* can be used as pioneer tree species for ecological restoration of copper tailing areas.

*R. acetosa* and *F. rubra* are perennial herbaceous plants with strong Cu adsorption capacities. Studies have indicated that *R. acetosa* is highly tolerant to complex metal contamination, particularly Cu accumulation potential (He et al., 2024). *F. rubra* is a grass species that demonstrates a high reproductive ability and strong tolerance to drought, cold, and metal toxicity (Karaca et al., 2018). As a root-accumulating species, *F. rubra* immobilizes metals in its roots, thereby effectively reducing the spread of contamination (Wyszkowska et al., 2022). Cuske et al. (2016) suggested that *F. rubra* can naturally colonize Cu tailings, with the Cu accumulation primarily in the roots, ranging from 298 mg/kg to 1,912 mg/kg, consistent with the findings of this study. Thus, planting *R. acetosa* and *F. rubra* in Cu tailings can effectively immobilize Cu in the root system, serving as a pioneer herbaceous species alongside trees to mitigate the spread of contamination during ecological restoration.

With advances in phytoremediation, single-species planting is insufficient for effective soil remediation in mining areas (Diallo et al., 2024). Mixed planting enhances soil microbial diversity and nutrient levels, helping maintain ecological balance (Lu et al., 2024). Vegetation restoration strategies can be tailored to the different levels of metal tolerance in plants. Predominant families, such as Asteraceae, Poaceae, and Polygonaceae, display a strong reproductive capacity and metal tolerance (Fu et al., 2017). Once pioneer species improve soil fertility, mixed planting of these tolerant species can be introduced to enrich vegetation community composition and increase ecosystem species diversity and stability in copper tailing areas.

### 4.3 Remediation ability of *Salix balfouriana* for metals in copper tailing soil

The efficiency of phytoremediation depends largely on the tolerance of plants to metals (Liu et al., 2010). Although hyperaccumulating plant species are generally better suited for phytoextraction, excluders have more advantages in phytostabilization. The current criteria for identifying hyperaccumulator plants involve two key standards: (1) the threshold content where the aboveground concentrations should exceed 100 mg/kg for Cd, 300 mg/kg for Cu, 1,000 mg/kg for Pb, and 3,000 mg/kg for Zn; and (2) enrichment and transfer coefficients greater than 1 (van der Ent et al., 2013; Manoel et al., 2014). None of the plant samples used in this study met the criteria. Membership function analysis revealed that *S. balfouriana* is the optimal candidate plant for the remediation of metal pollution in Dongchuan copper tailing soil. Previous studies have demonstrated that certain species of the genus *Salix* possess strong metal tolerance and accumulation capabilities, rendering them potential plants for the removal of soil metals (Yang et al., 2015). Eltrop et al. (1991) reported that *Salix caprea* exhibited strong tolerance to soil contaminated with high concentrations of Pb. Mohammad et al. (2012) discovered that *Salix purpurea* had an extremely high metal transfer ability in extremely complex pollution environments, with transfer coefficients of 4.72, 3.42, and 3.48 for Cu, Pb, and Zn, respectively. Moreover, Pavel et al. (2007) found that all seven *Salix* species could grow normally in soil with Cd, Pb, and Zn contents of 4.73 mg/kg, 1,158 mg/kg, and 180 mg/kg, respectively; and among them, *Salix smithiana* had the most potent ability to accumulate Cd and Zn in its aboveground parts. Currently, there are no reports on the application of *S. balfouriana* in the remediation of metal tailings. Given the remarkable metal adsorption capacity of *S. balfouriana* in Dongchuan copper tailings, it can be regarded as the prime choice of plant for pollution remediation in local mines. Nevertheless, its remediation efficiency and tolerance mechanisms deserve further exploration.

### 4.4 Correlation analysis between soil factors and plant metal content

Within certain limits, the concentrations of specific metals in the soil determine their corresponding adsorption levels by plants (Du et al., 2024). Metal concentration in the soil is a crucial factor influencing the metal adsorption capacity of plants (Mahdavian et al., 2022). Zu et al. (2004) discovered that the total concentration of Cd, Pb, Zn, and Cu in Pb–Zn tailings soil was in accordance with the order of metal concentration in plants, and the metal concentration in plants was positively correlated with the metal concentration in the soil, which is in accordance with the results of this study. Similarly, Karen et al. (2019) conducted a survey of 16 dominant plants in Armenian Cu tailings and identified a significant positive correlation between Cu concentrations in plant roots and total copper levels in the soil. Sławomir et al. (2024) reported that in the metal-contaminated areas, the concentrations of Pb, Zn, Cd, Ag, and Ti in dominant plants increased with increasing soil metal levels. According to other studies, within a specific range, the concentration of particular metals in the soil determines the adsorption level of plants, and the bioavailability of metals increases with an increase in the total metal concentration (Du et al., 2024; Xiao et al., 2022).

Metals often exist in multiple forms within the soil and determine their bioavailability of metals in soils (Feng et al., 2005). Studies have shown that the forms of metals in soil are affected by numerous factors, such as the total metal content, pH, organic matter content, redox potential, interaction between elements, and microorganisms (Zhang et al., 2014). This study further confirmed that soil pH and organic matter can alter the bioavailability of metals in the soil, thereby influencing the metal accumulation capacity of plants (Xu et al., 2022). A low pH usually enhances metal ion mobility, whereas a higher pH promotes the formation of stable metal–OM complexes, reducing metal bioavailability (Navarro et al., 2018). Research has indicated that, as soil pH decreases, the soil’s positive charge adsorption increases, enhancing the competitive adsorption of hydrogen ions and increasing free metal concentrations, thereby boosting plant uptake (Zeng et al., 2010). Conversely, a higher pH increases the stability of humic–metal complexes (Fu et al., 2019; Zhang et al., 2024). Humus is a key component of OM that facilitates the conversion of metals from their dissolved to organically bound forms in high-humus environments (Zhang et al., 2024). Under strongly oxidizing conditions, organically bound metals decompose, release them into the soil, and increase their mobility and bioavailability (Zhang et al., 2024). An increase in the bioavailability of metals in the soil, while it may increase the toxicity of the metals, can also promote the absorption of metals by plants, which is beneficial for phytoremediation (Rajkumar et al., 2009).

## 5 Conclusion

The soil in the Dongchuan Cu tailing area is heavily polluted with metals, primarily Cu, along with complex contamination from Zn, Pb, and Cd. A total of 29 dominant plant species were identified in this study. The metal concentrations in both plants and soil followed the trend Cu > Zn > Pb > Cd, with none of the species meeting the hyperaccumulator criteria. Among the identified plants, *L. leucocephala*, *R. acetosa*, *F. rubra*, and *S. balfouriana* exhibited the strong adaptability to the Cu tailing environment and relatively high metals content in their tissues, serving as the suitable pioneer species for ecological restoration. Membership function analysis confirmed that *S. balfouriana* was the optimal candidate for metal accumulation, highlighting its potential for ecological restoration in the Dongchuan Cu tailing area and its suitability for further research and broader applications.

## Data Availability

The original contributions presented in the study are included in the article/supplementary material. Further inquiries can be directed to the corresponding author.
